# Adaptive client participation mechanism for federated learning in heterogeneous vehicular networks

**DOI:** 10.1038/s41598-026-48453-y

**Published:** 2026-04-22

**Authors:** Wenhao Lin, Yang Zhou

**Affiliations:** 1 Department of Applied Mathematics, University of Washington, Seattle, 98195 WA USA; 2 Artificial Intelligence Laboratory, Shanghai University, Shanghai, 201109 China; 3 Water Technology Research Center, Chemical and Biomolecular Engineering Department, Henry Samueli School of Engineering and Applied Science, University of California, Los Angeles, Los Angeles, CA 90095 United States of America

**Keywords:** Engineering, Mathematics and computing

## Abstract

In vehicular networks, federated learning faces significant challenges due to resource heterogeneity, dynamic participation patterns, and intermittent connectivity among vehicles. Traditional client selection mechanisms often fail to consider the two-tier decision-making process inherent in vehicular network environments, where both central servers and individual vehicles must make participation decisions based on their respective constraints. Moreover, existing model aggregation algorithms typically assume fixed client participation and cannot adapt to the highly variable participation patterns unique to vehicular networks. This paper proposes a comprehensive vehicular federated learning framework with three key innovations. First, we introduce a strategy-driven adaptive client participation mechanism with a two-tier decision-making process that combines server-side reinforcement learning-based client selection with client-side autonomous participation decisions based on local resource thresholds. Second, we develop an incremental online policy learning algorithm based on Proximal Policy Optimization (IO-PPO) to address the data scarcity challenge in federated learning environments by enabling continuous learning from limited trajectory data. Third, we propose a dynamic client size-adaptive optimized model aggregation algorithm that adapts to different participation patterns while considering both current and historical client contributions. Our approach leverages a synergistic combination of reinforcement learning for adaptive decision-making, asynchronous federated learning principles for flexible participation, and graph-based modeling for capturing network topology effects. Extensive experimental results demonstrate that compared to existing methods, the proposed framework significantly improves learning efficiency, convergence stability, and model performance in realistic vehicular network scenarios.

## Introduction

The fast improvement of vehicular networks and the widespread adoption of connected vehicles have generated unprecedented amounts of data with immense potential for improving traffic management, autonomous driving, and intelligent transportation systems. However, traditional centralized machine learning approaches face significant challenges in this domain. These challenges arise from the sensitive nature of vehicular data, strict privacy regulations, and the distributed nature of vehicle-generated information^1^. Federated learning has emerged as a potential framework that facilitates collaborative model training among distant vehicles, safeguarding data privacy and minimizing communication overhead^2^..

Integrating federated learning in vehicular networks faces unique challenges that distinguish it from traditional federated learning scenarios. Vehicular network environments are characterized by high dynamics and heterogeneity. These include different computational capabilities, intermittent connectivity, and mobility patterns, which require innovative approaches to client participation and model aggregation^3^. Traditional federated learning algorithms assume relatively stable client participation and homogeneous resource availability. As a result, they often fail to deliver satisfactory performance in such dynamic environments^4^..

The diversity of computational and communication resources among cars constitutes a significant challenge in vehicular federated learning. Vehicles are equipped with different hardware configurations, ranging from basic onboard units to high-performance edge computing devices. These configurations result in vastly different capabilities for participating in federated learning tasks^5^. This resource heterogeneity, combined with the fluid characteristics of vehicular networks, creates challenging conditions. Vehicles regularly join and leave the network, making traditional client selection strategies inapplicable^6^.

Recent advances in asynchronous federated learning have demonstrated potential in resolving some of these challenges. These methods allow clients to participate in training at different times and frequencies, thereby adapting to irregular availability patterns common in vehicular networks^7^. Asynchronous methods enable more flexible participation patterns and can better handle the stochastic nature of vehicle availability. However, existing asynchronous federated learning methods still struggle to handle extreme resource heterogeneity and the need for intelligent client selection strategies that can adapt to rapidly changing network conditions.

Reinforcement learning has emerged as a powerful tool for addressing complex decision-making challenges in dynamic environments. This makes it particularly suitable for enhancing client selection and resource allocation in federated learning systems. Applying reinforcement learning to federated learning enables adaptive strategies. These strategies learn from past experiences and optimize long-term objectives rather than relying on static heuristics^8^. In vehicular network environments, reinforcement learning can effectively balance multiple competing objectives. These include minimizing communication latency, maximizing model accuracy, and ensuring fair participation among heterogeneous clients.

Graph Neural Networks (GNNs) have demonstrated exceptional capabilities in modeling complex relationships and dependencies in networked systems. This makes them naturally suited for vehicular network applications where vehicles, roadside units, and base stations form intricate communication topologies^9^. The spatial and temporal dependencies inherent in vehicular networks can be effectively captured using GNN-based methods. These methods model the dynamic relationships between network entities and their impact on federated learning performance.Furthermore, GNNs can facilitate better understanding of data quality and contribution patterns in vehicular network topologies.

Despite these technological advances, several fundamental challenges remain unresolved in vehicular federated learning. First, existing client selection mechanisms often fail to account for the two-tier decision-making process inherent in vehicular networks. In this process, both the central server and individual vehicles must make participation decisions based on their respective constraints and objectives^10^. Second, current model aggregation algorithms typically assume a fixed number of participating clients. They fail to adapt to the highly variable participation patterns characteristic of vehicular environments^11^.

The challenge of client participation in vehicular federated learning is further complicated by multiple competing factors. These include communication costs, computational capabilities, data quality, and privacy considerations^12^. Traditional approaches that rely on random selection or simple resource-based criteria often result in suboptimal performance and poor convergence properties^13^. Moreover, the dynamic nature of vehicular networks requires adaptive mechanisms that can respond to changing network conditions and client capabilities in real-time.

Another critical challenge lies in developing effective model aggregation strategies that can handle the uncertainty and variability in client participation. Standard federated averaging algorithms assume consistent participation patterns. They may exhibit poor convergence properties when the number and identities of participating clients vary significantly across training rounds^14^. This issue is particularly pronounced in vehicular networks. Vehicle availability is inherently unpredictable due to mobility patterns and varying mission requirements.

The integration of privacy-preserving mechanisms adds another layer of complexity to vehicular federated learning systems. Federated learning inherently provides some privacy protection by keeping data localized. However, vehicular applications often require additional privacy safeguards due to the sensitive nature of location and behavioral data^15^. Developing privacy-preserving client selection and model aggregation mechanisms that do not compromise learning efficiency remains an active area of research.

To address these multifaceted challenges, this paper proposes a comprehensive vehicular federated learning framework that incorporates three key innovations. First, we propose a strategy-driven adaptive client participation mechanism that employs a two-tier decision-making process. This combines server-side reinforcement learning-based client selection with client-side autonomous participation decisions based on local resource thresholds. Second, we introduce an incremental online policy learning algorithm based on Proximal Policy Optimization (IO-PPO). This algorithm addresses the data scarcity challenge in federated learning environments by enabling continuous learning from limited trajectory data. Third, we develop a dynamic client size-adaptive optimized model aggregation algorithm. This algorithm adapts to different participation patterns while considering both current and historical client contributions.

Our approach leverages a synergistic combination of three techniques: reinforcement learning for adaptive decision-making, asynchronous federated learning principles for flexible participation, and graph-based modeling for capturing network topology effects. The proposed framework is designed to handle the unique characteristics of vehicular networks. These include resource heterogeneity, dynamic participation patterns, and privacy requirements, while maintaining high learning efficiency and model performance.

The main contributions of this work can be summarized as follows: (1) A novel two-tier client participation mechanism that combines centralized intelligence with distributed autonomy to optimize resource utilization and training efficiency; (2) An innovative incremental learning approach that maximizes the utility of limited training data in federated learning scenarios; (3) A dynamic model aggregation algorithm that adapts to variable client participation while ensuring convergence stability; (4) A comprehensive evaluation that demonstrates the effectiveness of the proposed approach in realistic vehicular network scenarios.

## Related work

### Federated learning in vehicular networks

Federated learning has garnered considerable interest in vehicle networks for its capacity to facilitate collaborative learning while safeguarding data privacy. Early work by Li et al.^1^ established the theoretical foundations for federated optimization in heterogeneous networks. This work highlighted the unique challenges posed by non-Independent and Identically Distributed (non-IID) data distributions and varying computational capabilities among participants. Building upon this foundation, Mcmahan et al.^2^ proposed stochastic coded federated learning approaches that provide convergence and privacy guarantees. These approaches are particularly relevant for vehicular environments where communication reliability is a concern.

The application of federated learning to vehicular networks has been extensively studied by several researchers. Wang et al.^5^ introduced adaptive federated learning frameworks specifically designed for resource-constrained edge computing systems. These frameworks address the computational limitations inherent in vehicular environments. Their work demonstrated the importance of dynamic resource allocation and client selection strategies in maintaining learning efficiency. Similarly, Lim et al.^16^ provided a comprehensive survey of federated learning in mobile edge networks. They identified key challenges including intermittent connectivity, mobility patterns, and resource heterogeneity that are particularly pronounced in vehicular scenarios.

Recent advances in vehicular federated learning have focused on addressing specific challenges related to data quality and client reliability. Nguyen et al.^12^ explored the integration of blockchain technology with federated learning in edge computing environments. They proposed solutions for ensuring data integrity and participant accountability in vehicular networks. Their work highlighted the importance of trust mechanisms in distributed vehicular learning systems. Furthermore, Wei et al.^15^ developed differential privacy mechanisms for federated learning. These mechanisms provide formal privacy guarantees essential for protecting sensitive vehicular data. Recent research such as Yang et al.^17^ further explored privacy-preserving mechanisms in vehicular federated learning, proposing secure aggregation schemes based on homomorphic encryption.

### Client selection and participation mechanisms

Client selection represents one of the most critical challenges in federated learning systems, particularly in dynamic environments such as vehicular networks. Traditional client selection methods primarily focus on random sampling or resource-based criteria. However, these approaches often fail to account for the complex dynamics and heterogeneity present in vehicular environments.

Oh et al.^13^ proposed communication-efficient federated learning approaches using quantized compressed sensing. This approach indirectly addresses the client selection problem by reducing communication overhead and enabling participation of resource-constrained clients. Their work demonstrated that intelligent compression techniques could significantly expand the pool of viable participants in federated learning systems. Building upon this, Nishio et al.? developed resource availability-based client selection algorithms, considering device computational capabilities and battery status.

More sophisticated client selection strategies have been developed to address specific challenges of heterogeneous environments. Reddi et al.^14^ introduced adaptive federated optimization algorithms. These algorithms dynamically adjust client participation based on convergence requirements and system constraints. Their approach showed improved performance in scenarios with varying client capabilities and data distributions. Cho et al.^18^ proposed client contribution-based selection mechanisms, optimizing selection strategies by evaluating clients’ potential contributions to the global model.

The concept of strategic client participation has been explored by several researchers. Wang et al.^8^ developed incentive mechanisms for reliable federated learning. These mechanisms combine reputation systems with contract theory to encourage high-quality participation. Their work highlighted the importance of designing participation mechanisms that align individual client incentives with global learning objectives. Kang et al.^8^ further studied auction mechanism-based client selection methods, promoting active client participation through economic incentives.

Recent studies have revealed critical limitations in existing client selection and participation mechanisms for federated learning in dynamic environments. Research by Jain et al^19^. demonstrates that traditional incentive mechanisms relying on reputation systems and performance metrics like Mean Absolute Error (MAE) suffer from data quality decompensation issues, where redundant or low-quality client contributions can degrade global model performance despite high participation scores. Similarly, Moreno-Álvarez et al^20^. highlight that conventional auction-based and game-theoretic incentive mechanisms lack adaptability in dynamic environments and fail to ensure long-term client engagement, particularly when clients contribute heterogeneous data with varying quality levels. Furthermore, Jai^21^ identifies that existing approaches struggle with interpretability issues, making it difficult to understand why certain clients are selected and how their contributions affect model performance. These mechanisms also cannot effectively handle scenarios where multiple clients possess overlapping or redundant data samples, leading to inefficient resource utilization^22^. Our proposed reinforcement learning-based client selection mechanism addresses these fundamental gaps by introducing an explicit utility function that jointly considers data quality metrics, communication latency, and historical client reputation, combined with a two-tier decision-making process that enables both intelligent server-side selection and autonomous client-side participation decisions based on local resource constraints.

### Asynchronous federated learning

Asynchronous federated learning has arisen as a viable method to tackle the difficulties of irregular client availability and varying computational capabilities in dynamic networks. Xie et al.^7^ pioneered the development of asynchronous federated optimization algorithms. They demonstrated that allowing clients to participate at different times and frequencies could significantly improve system robustness and scalability.

Kang et al.^8^ extended asynchronous federated learning to geospatially distributed data scenarios. This is particularly relevant for vehicular networks where spatial and temporal data distributions vary significantly. Their work showed that asynchronous approaches could better accommodate the natural patterns of vehicle movement and availability.

The theoretical foundations of asynchronous federated learning have been further developed by several researchers. Kairouz et al.^4^ provided a comprehensive analysis of convergence properties in asynchronous federated systems. They identified conditions under which asynchronous approaches maintain convergence guarantees despite irregular participation patterns. Xie et al.^7^ studied delay issues in asynchronous federated learning, proposing time decay-based weight adjustment mechanisms.

Recent work has focused on optimizing asynchronous federated learning for specific application domains. Liu et al.^11^ developed FedVision, an online visual object detection platform. This platform leverages asynchronous federated learning principles to handle dynamic participation patterns in real-world deployment scenarios. Xu et al.^23^ proposed edge computing-oriented asynchronous federated learning architectures specifically designed for resource-constrained edge devices.

Despite significant progress in asynchronous federated learning, recent research has identified persistent efficiency challenges that limit its practical deployment in highly dynamic environments. Forootani and Iervolino^24^demonstrate that traditional Asynchronous Federated Learning (AFL) approaches face scalability limitations due to their implicit reliance on coordination mechanisms that assume relatively stable client availability patterns, resulting in significant delays and communication overhead in heterogeneous settings. Chen et al^25^. reveal that existing asynchronous frameworks struggle with model staleness accumulation and fail to maintain coherent global momentum across clients with highly variable participation patterns, particularly problematic in vehicular networks where vehicle availability fluctuates dramatically. Wang et al^26^. further show that conventional asynchronous aggregation strategies do not adequately adapt to dynamic changes in the number of participating clients across training rounds, leading to biased global models dominated by faster clients’ data distributions. Lu et al^27^. identify that stale updates in asynchronous systems can distort optimization dynamics and degrade model performance, especially under non-IID data distributions common in vehicular scenarios. Moreover, the survey by Dritsas and Trigka^28^ highlights that existing asynchronous methods in Internet of Things (IoT) environments often require more communication rounds to achieve target accuracy compared to synchronous approaches, negating their time efficiency advantages. Our proposed IO-PPO algorithm and dynamic client size-adaptive aggregation mechanism are specifically designed to address these limitations. First, IO-PPO enables truly flexible client participation through incremental online policy learning, without imposing implicit synchronization assumptions. Second, aggregation weights are adaptively adjusted based on both current client contributions and historical performance, thereby mitigating staleness-induced bias. Third, a momentum-based update strategy maintains convergence stability despite highly variable client participation patterns, significantly improving training efficiency in dynamic vehicular federated learning scenarios.

### Application of reinforcement learning in federated learning optimization

The application of reinforcement learning to federated learning optimization has gained significant attention due to its capacity to manage intricate multi-objective optimization challenges in dynamic environments. Early work by Wang et al.^29^ demonstrated the effectiveness of reinforcement learning approaches for optimizing federated learning on non-IID data. Their results showed that adaptive strategies could significantly outperform static client selection methods.

Xu et al.^30^ developed federated reinforcement learning frameworks for resource allocation in Vehicle-to-Everything (V2X) networks, demonstrating how reinforcement learning could be applied to optimize resource utilization in vehicular federated learning systems. Their work highlighted the importance of dynamic resource allocation strategies in vehicle-to-everything communication environments. Chen et al.^31^ further explored multi-agent reinforcement learning applications in federated learning, proposing distributed decision-making frameworks.

More recent developments have focused on multi-agent reinforcement learning approaches for federated systems. Krouka et al.^32^ proposed collaborative reinforcement learning frameworks. In these frameworks, multiple agents work together to optimize global learning objectives while maintaining local autonomy. Their approach is particularly relevant for vehicular networks that require both centralized coordination and distributed decision-making. Sagar et al.^33^ developed hierarchical adaptive federated reinforcement learning algorithms for efficient resource allocation and task scheduling in IoT networks, proposing a primal-dual update based deep deterministic policy gradient method for effective resource management.

The integration of reinforcement learning with federated learning has also been explored for specific optimization objectives. Li et al.^22^ developed reinforcement learning algorithms for minimizing communication costs in federated learning systems, demonstrating significant improvements in bandwidth utilization and training efficiency. He et al.^34^ proposed reinforcement learning-based adaptive aggregation strategies, dynamically adjusting aggregation weights based on clients’ historical performance.

### Graph neural networks in vehicular networks

Graph Neural Networks have shown exceptional promise for modeling complex relationships in networked systems, making them particularly suitable for vehicular network applications including transportation and communication networks. Wu et al.^9^ provided a comprehensive survey of graph neural networks, establishing the theoretical foundations for their application in various domains.

Shao et al.^35^ proposed a novel GNN-based framework for task offloading and resource allocation in dynamic vehicular networks, demonstrating that GNNs could effectively process variable-dimensional environmental information and make scalable decisions that adapt to network size changes. Their work showed that GNNs could effectively capture both structural and feature-based information in dynamic vehicular scenarios. Jiang et al.^36^ proposed graph convolutional networks, providing a foundational framework for spatial relationship modeling in vehicular networks.

The application of GNNs to vehicular networks has been explored from multiple perspectives. Zhang et al.^37^ developed graph-based approaches for vehicular network traffic prediction. Their work showed that GNNs could effectively model spatial and temporal dependencies in vehicular data. Their work demonstrated the potential for GNNs to enhance data quality assessment and client contribution evaluation in federated learning scenarios. Zhang et al.^38^ further studied the application of graph neural networks in vehicular network routing optimization.

Recent advances have focused on dynamic graph neural networks that can adapt to changing network topologies. Sadid et al.^39^ proposed temporal graph neural networks for vehicular networks, addressing the challenge of modeling time-varying relationships between network entities. Their approach provides insights into how GNNs could be integrated with federated learning systems to better understand client relationships and optimize participation strategies. Zhang^40^ developed dynamic graph convolutional networks with temporal representation learning for traffic flow prediction, introducing a temporal graph convolution block that treats historical time slots as graph nodes to capture flexible global temporal dependencies and enhance understanding of current traffic conditions.

### Model aggregation in dynamic environments

Traditional federated learning relies on simple averaging techniques for model aggregation, which may not be optimal for dynamic environments with varying client participation patterns. The challenge of developing adaptive aggregation strategies has been addressed by several researchers.

McMahan et al.^2^ established the foundational FedAvg algorithm, which uses weighted averaging based on client data sizes. However, subsequent research has shown that this approach may not be optimal for heterogeneous and dynamic environments typical of vehicular networks. Li et al.^1^ proposed the FedProx algorithm, addressing system heterogeneity by adding regularization terms.

Advanced aggregation strategieshave been developed to tackle certain issues in dynamic federated learning systems. Li et al.^6^ proposed federated learning approaches that account for system heterogeneity. They developed aggregation methods that consider both data quality and client reliability. Their work demonstrated the importance of adaptive weighting schemes in maintaining learning performance under varying participation patterns. Wang et al.^5^ further studied dynamic weight adjustment mechanisms based on client contributions.

The concept of personalized aggregation has been explored by several researchers. Mansour et al.^41^ developed three federated learning approaches that balance global model performance with personalization requirements. Their work is particularly relevant for vehicular applications where different vehicles may have distinct operational patterns and data characteristics.

Recent work has focused on aggregation algorithms that can handle extreme variations in client participation. Fallah et al.^42^ proposed personalized federated learning approaches that maintain performance even when client participation varies dramatically across training rounds. Their work provides insights into designing robust aggregation mechanisms for highly dynamic environments. Karimireddy et al.^43^ developed the SCAFFOLD algorithm, addressing client drift issues through control variables.

### Privacy and security in vehicular federated learning

Privacy and security considerations are paramount in vehicular federated learning systems due to the sensitive nature of location and behavioral data. The development of privacy-preserving mechanisms has been an active area of research.

Differential privacy has been extensively studied as a mechanism for protecting client privacy in federated learning. Shukla et al.^44^ introduced federated learning with differential privacy for healthcare applications, demonstrating that privacy protection could be achieved without significantly compromising learning performance by injecting statistical noise into model updates. Their work showed that Federated Learning (FL) combined with Differential Privacy (DP) achieves high accuracy while ensuring strong privacy preservation.Zhang et al.^45^ further developed adaptive differential privacy mechanisms for asynchronous federated learning in aerial-aided edge computing, proposing adaptive gradient clipping strategies and game-theoretic incentive schemes to enhance the balance between model utility and privacy.

Secure aggregation techniques have been developed to protect against various attack vectors in federated learning systems. Ratnayake et al.^46^ proposed privacy-preserving federated learning with intermediate-level model sharing, introducing a hybrid weighting mechanism for model aggregation that integrates sample size and test accuracy while addressing privacy vulnerabilities at central servers. Their approach is particularly important for vehicular networks where multiple parties may have different trust relationships. Li et al.^47^ developed cryptography-based secure multi-party computation schemes.

The challenge of maintaining privacy while ensuring data quality has been addressed by several researchers. Khalaf et al.^48^ developed hybrid differential privacy approaches for secure and reliable cross-IoT platform knowledge sharing, introducing novel methods where users collaborate to train shared models with gradient-based contributions adjusted according to trust levels. Zhao et al.^49^ proposed homomorphic encryption-based privacy-preserving federated learning frameworks.

Recent work has focused on privacy-preserving client selection mechanisms. Mohammadi et al.^50^ integrated federated learning and differential privacy for secure anomaly detection in smart grids, proposing methods to balance noise scale and model accuracy while maintaining strong privacy guarantees. Their work is particularly relevant for vehicular scenarios where privacy protection is essential for sensitive operational data. So et al.^51^ studied Byzantine fault tolerance mechanisms in federated learning, ensuring system robustness in the presence of malicious participants.

## Methods

### System model

**Fig. 1:**
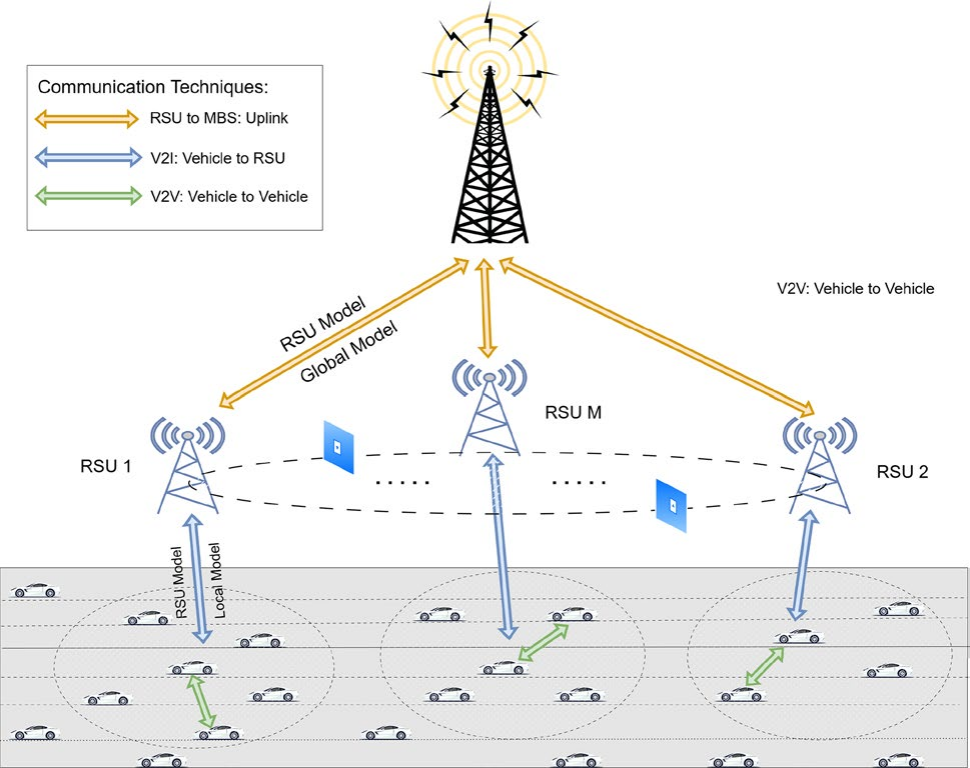
Data sharing in vehicular networks.

In Vehicular Networks, vehicles, RSUs (Roadside Units), and MBSs (Macro Base Stations) are considered key components of the vehicular network infrastructure (see Fig. 1). Let $V = \{v_i\}$ be the set of vehicles. Comparing to the vehicles in the network, the MBSs have superior number of resources in terms of computation and communication. The communication infrastructure supports three types of links as illustrated in Fig. 1. First, V2V communication enables direct vehicle-to-vehicle data exchange within communication range, typically utilizing IEEE 802.11p or Cellular Vehicle-to-Everything (C-V2X) protocols with bandwidth $B_{v2v}$ and average latency $\tau _{v2v}$. Second, Vehicle-to-Infrastructure (V2I) communication (specifically V2R) facilitates uplink and downlink transmission between vehicles and RSUs, with available bandwidth $B_{v2i}$ and latency $\tau _{v2i}$. Third, RSU-to-MBS uplink provides high-capacity backhaul connectivity with bandwidth $B_{backhaul}$.

We assume that V2V links experience higher variability due to vehicle mobility, while V2I links provide more stable connectivity within RSU coverage zones. Vehicles in the network *V* have limited resources. These resources are contributed by on-board units and portable devices within the vehicle. RSUs are usually supplied with Multi-access Edge Computing (MEC) servers and possess specific computing and storage availabilities. They establish uplink communication to connect to the MBSs and downlink communication to link to vehicles within their coverage area. The transmissions of data between RSUs and vehicles or within vehicles in the network are described as Vehicle-to-RSU (V2R) or Vehicle-to-Vehicle (V2V) communication.

Vehicle $v_{req}$ requests to share certain data (e.g., for navigation or traffic prediction). It collaborates based on computational results *Res* from its data sharing request *Req* with respect to the shared data $\mathcal {D}$. This so-called sharing process can be understood as a computational job. Suppose that $V_I = \{v_1, v_2, \ldots , v_n\}$ are vehicles in the network, and the corresponding datasets are $\mathcal {D} = \{D_1, D_2, \ldots , D_n\}$. The aim of the computational job is to form a data model $\mathcal {M}$ based on $\mathcal {D}$ to respond to the data sharing request of $v_{req}$.

### Strategy-driven adaptive client participation mechanism

**Fig. 2:**
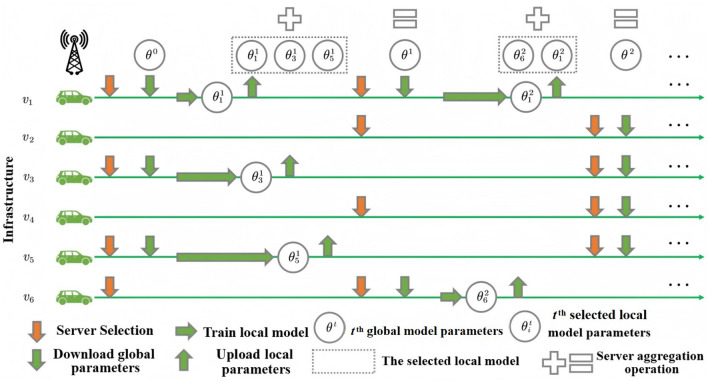
Federated Learning Training Process Based on Client Participation Mechanisms.

To balance the resource heterogeneity among clients in federated learning, this paper proposes a strategy-driven client participation mechanism. The federated learning training process based on this mechanism is shown in Fig. 2. At communication round *t*, the server collects information on all clients’ current computing and communication resources before initiating communication. Based on this information, the server chooses a subset $V_s^t$ from the entire set of vehicle clients *V*. The selected clients $v_i \in V_s^t$ then make a secondary decision based on their resource status. They determine whether to participate in this round before communication begins. After the clients that do not meet the requirements opt out, the final subset of clients participating in this round is $V_s^t \subseteq V_s^t$.

In the server-side decision-making process, we employ a deep reinforcement learning model. This model formulates a client selection strategy that balances communication delay and training effectiveness. Specifically, we design a reward function based on two factors: current resource heterogeneity within the system and the aggregated model’s performance. This reward function is then used to train the deep reinforcement learning model to identify the participating clients in the federated learning communication rounds.

On the client side, each client maintains a personalized resource threshold locally. If selected by the server, a client decides whether to participate based on its resource availability. Participation occurs only if current computing and communication resources exceed local thresholds. In practical scenarios, this threshold can be determined by historical resource monitoring results. For example, if the current available computing resources exceed the 30th percentile of the historical computing resource measurements, the computing resources are considered sufficient for training.

If fewer than 2 clients participate during the learning process, federated learning conditions are not met. In this case, the server re-collects the latest client status. Both server and clients make new decisions until federated learning conditions are satisfied.

The total latency $\delta _{total,i}^{t}$ of client *i* during the *t*-th round of communication is defined as:

1 $$\begin{aligned} \delta _{total,i}^{t} = \delta _{calculation,i}^{t} + \delta _{transmission,i}^{t} \end{aligned}$$

where $\delta _{calculation,i}^{t}$ and $\delta _{transmission,i}^{t}$ means the computation latency and communication latency of client *i* in the *t*-th round of communication, respectively.

To facilitate the study, the computation latency $\delta _{calculation,i}^{t}$ is estimated using the ratio of the computational workload required for training to the client’s currently available computational power, defined as:

2 $$\begin{aligned} \delta _{calculation,i} = \frac{N_i^t O}{\zeta _i^t \phi _i} \end{aligned}$$

where $N_i^t$ represents the amount of training data for client *i* in the *t*-th round of communication, *O* represents the computational workload to finish processing one unit of data, $\zeta _i^t$ indicates the computational power discount factor of client *i* in the *t*-th round, and $\phi _i$ denotes the theoretical peak computational power of the client’s local hardware.

The communication latency $\delta _{transmission,i}^{t}$ consists of the downlink latency $\delta _{download,i}^{t}$ and uplink latency $\delta _{upload,i}^{t}$, defined as:

3 $$\begin{aligned} \delta _{transmission,i}^{t} = \delta _{download,i}^{t} + \delta _{upload,i}^{t} \end{aligned}$$

where $$\delta _{download,i}^{t} = \frac{\varrho (\theta ^{t-1})}{b_{down,i}^{t}}$$, $$\delta _{upload,i}^{t} = \frac{\varrho (\theta _i^t)}{b_{up,i}^{t}}$$

where $\varrho (\theta ^{t-1})$ and $\varrho (\theta _i^t)$ represent the size of the global model parameters in the *t*-th round of communication and the size of the local model parameters of client *i* in the *t*-th round, respectively. $b_{down,i}^{t}$ and $b_{up,i}^{t}$ denote the available downlink and uplink bandwidths for client *i* in the *t*-th round, respectively.

To objectively assess the data quality of the client while ensuring the client’s privacy, the data quality metric $Q_i^t$ of client *i* is expressed as:

4 $$\begin{aligned} Q_i^t = \frac{1}{t} \sum _{j=1}^{t} \overline{q}_i^j\cdot \textbf{1}(v_i \in V_s^j) \end{aligned}$$

where *t* refers to the round of communication, $\textbf{1}(\cdot )$ is an indicator function, if and only if $t = 1$.

Reinforcement learning can be modeled as a Markov Decision Process (MDP), defined as Eq. (5):

5 $$\begin{aligned} \mathcal {M} = (\mathbb {S}, \mathbb {A}, \mathcal {P}, \rho , r) \end{aligned}$$

where $\mathbb {S}$ represents the state space, $\mathbb {A}$ represents the action space, $\mathcal {P}(s^+|s,a)$ denotes the probability of transitioning from state *s* to state $s^+$ after taking action *a*, $\rho (s)$ indicates the probability of the initial state being *s*, and *r*(*s*, *a*) represents the reward obtained by performing action *a* in state *s*.

In this paper, it is assumed that there are *M* clients in the system. The state information $s_t$ ($s_t \in \mathbb {S}$) in communication round *t* includes the data quality metrics $Q^t = [Q_1^t, Q_2^t, \ldots , Q_M^t]$, the amount of data $N^t = [N_1^t, N_2^t, \ldots , N_M^t]$ intended for training, the available computational power $\phi ^t = [\zeta _1^t \phi _1, \zeta _2^t \phi _2, \ldots , \zeta _M^t \phi _M]$, the available downlink bandwidth $b_{down}^t = [b_{down,1}, b_{down,2}, \ldots , b_{down,M}]$, and the available uplink bandwidth $b_{up}^t = [b_{up,1}, b_{up,2}, \ldots , b_{up,M}]$ for all *M* clients $(n \in V)$ before the start of the communication round *t*.

6 $$\begin{aligned} s_t = (Q^t, N^t, \phi ^t, b_{down}, b_{up}) \end{aligned}$$

For the action space, before each communication round, the server selects *n* of *M* clients. Therefore, the size of the action space is $C_M^n$. To decrease the model complexity and accelerate the convergence of model training, the input to the action model $A(\psi _a)$ is defined as *s* and the output is the selection probabilities for the *M* clients. In each decision round, the top *n* clients are selected based on these output probabilities, and their indices serve as the client indices for the server’s selection decision as well. The size of the action space is reduced to *M*, where $\psi _a$ represents the parameters of the action model. The action information $a_t'$ ($a_t' \in \mathbb {A}$) is defined as the selection decision on the server side, expressed as Eq. (7):

7 $$\begin{aligned} a_t' = \{i | i = 0, 1, \ldots , M-1, \text { and } \textbf{1}(v_i \in V_s^{t'}) = 1\} \end{aligned}$$

The actual client participation result after the clients’ secondary decision-making is defined as a set of actions $a_t$, expressed as Eq. (8):

8 $$\begin{aligned} a_t = \{i | i = 0, 1, \ldots , M-1, \text { and } \textbf{1}(v_i \in V_s^t) = 1\} \end{aligned}$$

By designing a client utility function, we can objectively assess each participating client’s contribution to the aggregated global model in the current communication round. $\forall v_i \in V_s^t$ The utility is calculated as:

9 $$\begin{aligned} U_i^t = {\left\{ \begin{array}{ll} \mu \left( 1 - \frac{\max (Q^t) - Q_i^t}{\max (Q^t)}\right) + \frac{v}{\delta _{total,i}^{t}}, & \text {if } ACC> \text {previous } ACC \\ \mu {\left( \frac{\max (Q^t) - Q_i^t}{\max (Q^t)}\right) }^2 + \frac{v}{\delta _{total,i}^{t}}, & \text {if } ACC \le \text {previous } ACC \end{array}\right. } \end{aligned}$$

where $\mu$ and *v* are the hyper parameters used to adjust the weights between data quality and latency. *ACC* and previous *ACC* represent the Top 1 accuracy of the current global model and the previous global model, respectively.

Based on the client’s utility, a client reputation mechanism is designed. This mechanism calculates the reputation value $\psi _i^t$ for each client based on its historical utility. The reputation value $\psi _i^t$ of client $v_i$ ($v_i \in V_s^t$) in communication round *t* is calculated as:

10 $$\begin{aligned} \psi _i^t = \lambda \psi _i^{t-1} + (1-\lambda ) U_i^t \end{aligned}$$

where $\lambda$ is the momentum update weight hyperparameter. Then, the reward function $r_t(s_t, a_t)$ in communication round *t* is defined as:

11 $$\begin{aligned} r_t(s_t, a_t) = \sum _{v_i \in V_s^t} \psi _i^t \end{aligned}$$

It is worth mentioning that this reward function calculates the cumulative reputation value of the subset of clients that participate in communication after the clients’ secondary decision-making.

In the current round *t*, executing action $a_t$ in state $s_t$ transitions to the next state $s_{t+1}$. This transition can be inferred from Eq. (11). The interaction trajectory $\tau _t = (s_1, a_1, s_2, a_2, \ldots , s_t, a_t)$ between the agent and the environment in this study describes the federated learning training process up to the current round *t*.

### Incremental online policy learning based on PPO

Before introducing the incremental online policy learning algorithm, we formally define the state and action spaces of our reinforcement learning framework.

State space: At each communication round *t*, the state $s_t \in \mathcal {S}$ captures the current status of all clients, consisting of data quality metrics $Q^t$, training data amounts $N^t$, computational power $\phi ^t$, and bandwidth availability ($b_{down}^t$, $b_{up}^t$), as defined in Eq. 6.

Action space: The action $a_t' \in \mathcal {A}$ represents the server’s client selection decision (Eq. 7). To reduce the selection complexity from $C_M^n$ to *M*, the action model $\mathcal {A}(s; \psi _a)$ outputs a selection probability for each client, and the top *n* candidates are chosen. The final participating set $a_t$ (Eq. 8) is then determined after each client applies its local resource-based decision.

In policy gradient-based deep reinforcement learning, policy $\pi (\cdot | \cdot ; \psi _a)$ governs the behavior of the agent in a specific environment. $\pi (a'|s; \psi _a)$ denotes the possibility of taking action $a'$ in state *s*, that is, when $a'$ serves as the output node index set of the model $\mathcal {A}(s; \psi _a)$, the value set corresponds to the output nodes of this index set. Since $a \subseteq a'$, *a* still depends on *s*, and its policy probability is influenced by $\psi _a$. Therefore, the occurrence probability of the trajectory $\tau _t$, which depends on $\pi (\psi _a)$, can be expressed as:

12 $$\begin{aligned} q(\tau _t; \psi _a) = \rho (s_0) \prod _{i=1}^{t-1} \pi (a_i|s_i; \psi _a) \prod _{i=1}^{t-1} \mathcal {P}(s_{i+1}|s_i, a_i) \end{aligned}$$

where the cumulative reward of a single trajectory $\tau _t$ is defined as Eq. (13):

13 $$\begin{aligned} R(\tau _t) = \sum _{i=1}^{t} \gamma ^{i-1} r(s_i, a_i) \end{aligned}$$

where $\gamma$ is the reward discount factor, and its exponent is the number of training rounds *t* minus 1. The deep reinforcement learning process at this point is defined as maximizing the objective function:

14 $$\begin{aligned} \arg \max _{\psi _a} \mathcal {J}(\psi _a) = \mathbb {E}_{\tau \sim q(\cdot |\psi _a)} [R(\tau )] \end{aligned}$$

This process identifies the optimal parameters $\psi _a^*$ such that policy $\pi (\psi _a^*)$ maximizes the average cumulative reward. Since gradient descent is standard in deep learning optimization, the objective is equivalently reformulated as a minimization problem. Therefore, in the practical implementation, policy gradient-based deep reinforcement learning is defined as solving the objective function:

15 $$\begin{aligned} \arg \min _{\psi _a} f(\psi _a) = -\mathcal {J}(\psi _a) \end{aligned}$$

When applying the deep reinforcement learning model to the federated learning client selection system, the trajectory data available for training the policy model is very limited. For federated learning with *T* communication rounds, only one trajectory with *T* time steps is generated. After data reuse, *T* trajectories become available for training. Two consecutive trajectories $\tau _i$ and $\tau _{i+1}$ ($i \in [1, T-1]$) differ by one time step, where $\tau _i$ is a prefix of $\tau _{i+1}$.

To improve data utilization, this paper proposes a deep reinforcement learning algorithm built on Proximal Policy Optimization (PPO), named ”Incremental Online Policy Learning based on PPO, IO-PPO.” Specifically, after the completion of federated communication in the current round *t*, online reinforcement learning training is performed using the current trajectory data $\tau _t$. Since the model is trained incrementally with each additional time step of trajectory data, the training process is a form of incremental learning.

Define the state value function $V(\psi _v)$ and train it using the Mean Squared Error (MSE) loss function as in Eq. (13). The training process in the current communication round *t* is described by the following minimization problem:

16 $$\begin{aligned} \arg \min _{\psi _a} f(\psi _v) = \frac{1}{t-1} \times \sum _{i=1}^{t-1} (V(s_i; \psi _v) - (r(s_i, a_i) + \gamma V(s_{i+1}; \psi _v)))^2 \end{aligned}$$

where $\psi _v$ represents the parameters of the state value model, and $t> 1$.

Based on Generalized Advantage Estimation (GAE), the truncated generalized advantage estimation vector $\Lambda (\tau _t)$ for the trajectory in communication round *t* is defined as:

17 $$\begin{aligned} \Lambda (\tau _t) = [A_1, A_2, \ldots , A_t] \end{aligned}$$

where $$A_k = \sum _{i=k}^{t} (\gamma \lambda _A)^{i-k} (r(s_i, a_i) + \gamma V(s_{i+1}; \psi _v) - V(s_i; \psi _v))$$

$\lambda _A$ is a hyper parameter in GAE used to balance bias and variance.

The importance sampling ratio vector $\mathcal {R}(\tau _t)$ for communication round *t* is calculated as follows:

18 $$\begin{aligned} \mathcal {R}(\tau _t) = [\delta _1, \delta _2, \ldots , \delta _t] \end{aligned}$$

where $\delta _k = \frac{\pi (a_k|s_k; \psi _{a,new})}{\pi (a_k|s_k; \psi _{a,old})}$. $\psi _{a,new}$ and $\psi _{a,old}$ represent the latest action model parameters and the old action model parameters before the current round of reinforcement learning training, respectively. After $\psi _a$ is trained for one or more epochs, $\psi _{a,new}$ and $\psi _{a,old}$ become distinct.

At this point, the deep reinforcement learning process in the current round *t* is redefined to maximize the objective function $\mathcal {J}_t(\psi _a)$:

19 $$\begin{aligned} \arg \max _{\psi _a} \mathcal {J}_t(\psi _a) = \min (\mathcal {R}(\tau _t) \Lambda (\tau _t)^{\top }, \text {clip}(\mathcal {R}(\tau _t), 1-\epsilon , 1+\epsilon ) \Lambda (\tau _t)^{\top }) \end{aligned}$$

where ${}^{\top }$ is the transpose of the vector $A(\tau _t)$. The clip function $\text {clip}(\cdot )$ applies to each element $\delta _k$ of $\mathcal {R}(\tau _t)$ to constrain it within the range $[1-\epsilon , 1+\epsilon ]$, where $\epsilon \in (0, 1)$ is a hyperparameter. Substituting the modified $\mathcal {J}_t(\psi _a)$ back to Eq. (15) yields the minimization objective function $f_t(\psi _a)$ for round *t*:

20 $$\begin{aligned} \arg \min f_t(\psi _a) = -\mathcal {J}_t(\psi _a) \end{aligned}$$

The detailed process of the proposed algorithm is given in Algorithm 1.

**Algorithm 1:**
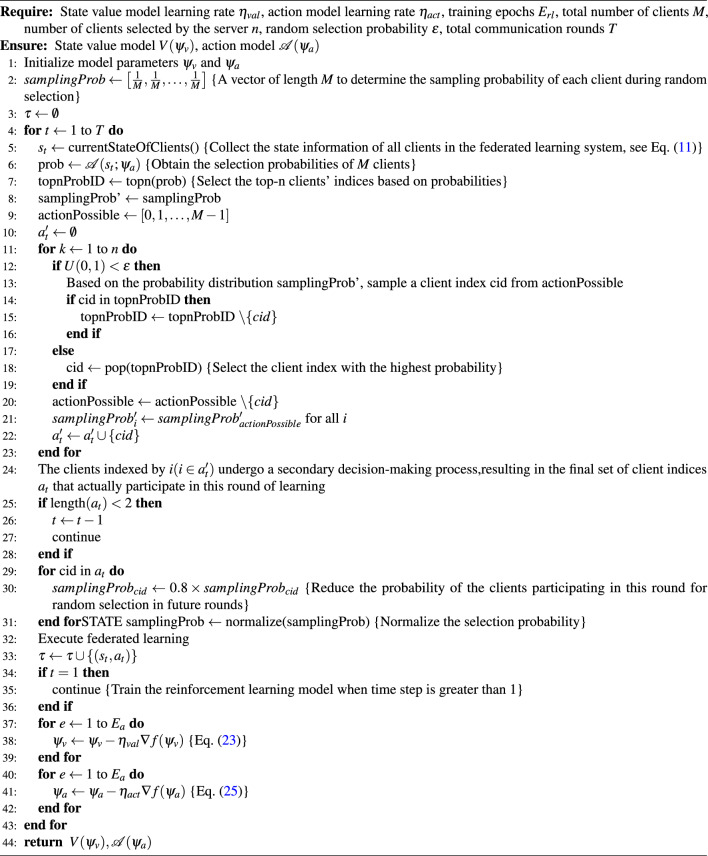
Incremental Online Policy Learning based on PPO, IO-PPO

### Dynamic client size-adapted optimized model aggregation algorithm

In the proposed client participation mechanism (Section “Methods”), the server sets an expected number of participating clients. However, the actual number remains uncertain due to clients’ autonomous decision-making. This dynamic participation pattern can considerably affect training stability and convergence. The impact is especially significant when client numbers fluctuate substantially. To solve this problem, this section optimizes the model aggregation algorithm in IO-PPO to adapt to the dynamically changing client size.

At training round *t*, a varying number of local models may be sent to the server from the client set $V_{s}^{t'}$. Let this data be denoted as $n_t$, i.e., set $|V_s^t| = n_t$. Accordingly, the aggregation weight vector is defined as $w^t = [w_0^t, w_1^t, \ldots , w_{n_t}^t]$. Each client $v_i$’s model parameter $\theta _i^t$ is obtained by training on its local data and applying the updates on the global model parameters from the previous round $\theta ^{t-1}$. In theory, the aggregation weight $w_i^t$ should reflect the incremental effective information provided by client $v_i$ to the model $f(\theta ^{t-1})$ in this training round.

In federated learning with dynamic client size, the optimized aggregation algorithm must consider multiple information sources. It should account for current participants’ contributions and also the historical contributions from all *M* clients across previous training rounds. This dual consideration ensures quick and smooth convergence. However, quantifying a single client’s contribution to the global model is challenging. This difficulty arises because global model parameters are updated through multiple rounds of nonlinear computation with different clients’ data.

The local model weight $w_i^t$ of client $v_i$ is determined by the mean of the validation results $\overline{q}_i^t$ on the link with parameters $\theta _i^t$. After multi-party validation, $\overline{q}_i^t$ can somewhat measure the amount of effective information that model $f(\theta _i^t)$ has obtained from the local data of client $v_i$ in this round of training. As long as the global model remains unconverged, any round of local training can be seen as a process of extracting effective information from the clients’ local data, which is heavily affected by the quality of the local data.

Based on these considerations, two fundamental principles are proposed to optimize the aggregation weights. First, $w_i^t$ should reflect the current-round link validation mean $\overline{q}_i^t$ of each participating client $v_i$, ensuring that clients providing more effective information receive higher weights. Second, $w_i^t$ should also incorporate the historical link validation performance of $v_i$ across all previous rounds, capturing its cumulative contribution to the global model.

The first principle ensures that the aggregation weights are fairly distributed so that clients with more effective information will be assigned higher weights to the global model. The second principle further accounts for the historical performance of clients and the potential contribution of clients who did not participate in the current training round to the soon-to-be-updated global model parameters $\theta ^t$.

By using this method, the aggregation algorithm reflects both real-time training dynamics and long-term data contributions, enhancing the stability and convergence speed of the global model. This strategy, which combines both real-time and historical

**Fig. 3:**
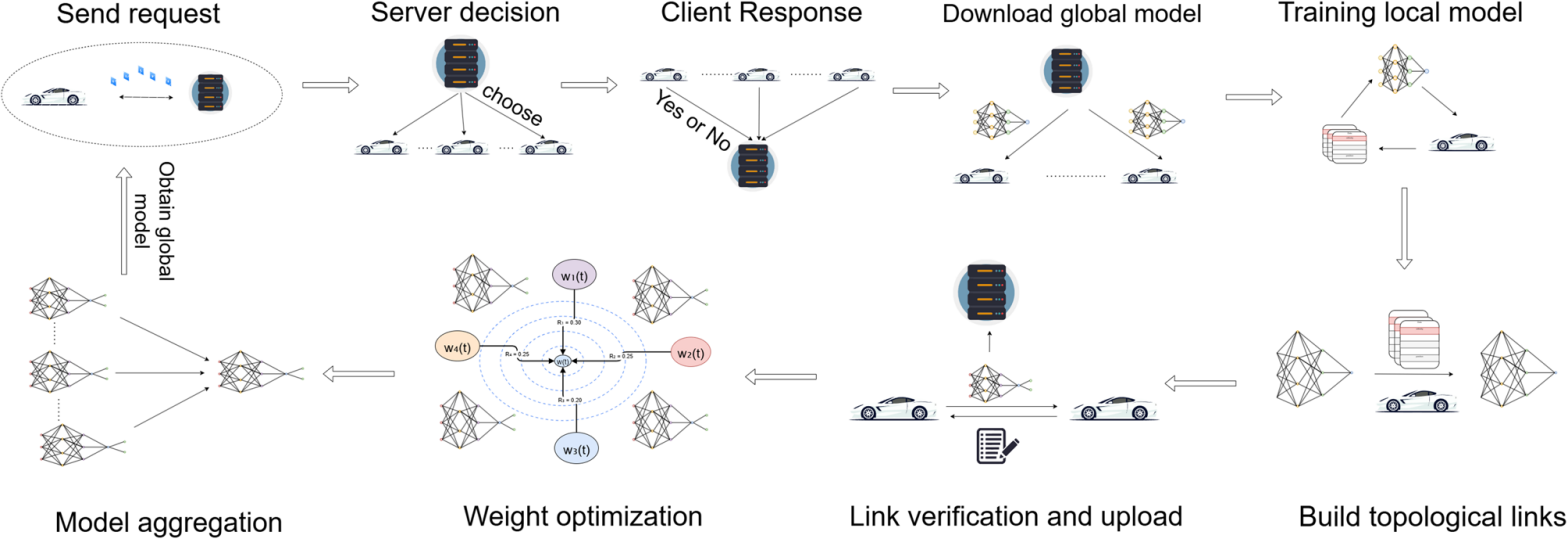
Proposed method workflow showing the federated learning training process with dynamic client participation.

data, significantly improves traditional model aggregation methods, effectively addressing the uncertainty and variability of client participation.

Let $\beta _t$ be the normalized contribution vector of the $n_t$ participating clients to the global model $f(\theta ^t)$ in the *t*-th round of federated learning:

21 $$\begin{aligned} \beta _t = \begin{bmatrix} \frac{\exp (\overline{q}_0^t)}{\sum _{j=0}^{n_t-1} \exp (\overline{q}_j^t)},&\frac{\exp (\overline{q}_1^t)}{\sum _{j=0}^{n_t-1} \exp (\overline{q}_j^t)},&\ldots ,&\frac{\exp (\overline{q}_{n_t-1}^t)}{\sum _{j=0}^{n_t-1} \exp (\overline{q}_j^t)} \end{bmatrix} \end{aligned}$$

where $\beta _{t-1}$ is the normalized contribution vector of the $n_t$ participating clients to the global model $f(\theta ^{t-1})$ in the *t*-th round of federated learning:

22 $$\begin{aligned} \beta _{t-1} = \begin{bmatrix} \frac{\exp (Q_0^{t-1})}{\sum _{j=0}^{n_t-1} \exp (Q_j^{t-1})},&\frac{\exp (Q_1^{t-1})}{\sum _{j=0}^{n_t-1} \exp (Q_j^{t-1})},&\ldots ,&\frac{\exp (Q_{n_t-1}^{t-1})}{\sum _{j=0}^{n_t-1} \exp (Q_j^{t-1})} \end{bmatrix} \end{aligned}$$

where $t> 1$ and $Q_i^{t-1}$ are the data quality metrics calculated using Eq. (9), with the subscript *i* indicating the *i*-th participating client.

At this point, when $t> 1$, the optimization of vector $w^t$ can be described by the following minimization problem:

23 $$\begin{aligned} \begin{aligned} \arg \min _{w^t} \, f(w^t)&= \left\| \xi _t \odot w^t - \beta _{t-1} \right\| _2^2 + \left\| (1 - \xi _t) \odot w^t - \beta _t \right\| _2^2 \\ \text {s.t.} \quad&\sum _i^{n_t} w^t_i = 1, \\&w^t_i \ge 0, \quad i = 0, 1, \dots , n_t - 1 \end{aligned} \end{aligned}$$

where $\xi _t$ is the weight parameter for global model momentum updates. If the optimal solution of Eq. (23) is obtained as $w^{t*}$, the model aggregation algorithm for the *t*-th round of federated learning ($t> 1$) is expressed as:

24 $$\begin{aligned} \theta ^t = \xi _t \theta ^{t-1} + (1-\xi _t) \sum _{i=0}^{n_t-1} w_i^{t*} \theta _i^t \end{aligned}$$

Given the uncertainty of client size, the aggregation step size $1-\xi _t$ of the global model should stay variable during each round of federated learning instead of applying a fixed step size. Therefore, this paper defines $\xi _t$ as an adaptive parameter that adjusts according to the current model’s convergence level:

25 $$\begin{aligned} \xi _t = \frac{1}{t-1} \sum _{i=1}^{t-1} \frac{1}{n_i} \sum _{k=0}^{n_i-1} \overline{q}_k^i \end{aligned}$$

It can be seen from Eq. (25) that when the model $f(\theta ^{t-1})$ generally achieves high validation results in historical training rounds, it indicates that the model $f(\theta ^{t-1})$ has sufficiently acquired effective information. Accordingly, when historical validation results are high, the aggregation step size $1-\xi _t$ is reduced; otherwise, it is increased.

To solve the minimization problem in Eq. (23), this paper introduces the gradient descent method to approximate the optimal solution $w^{t*}$. The detailed process is explained in Algorithm 2.

**Algorithm 2:**
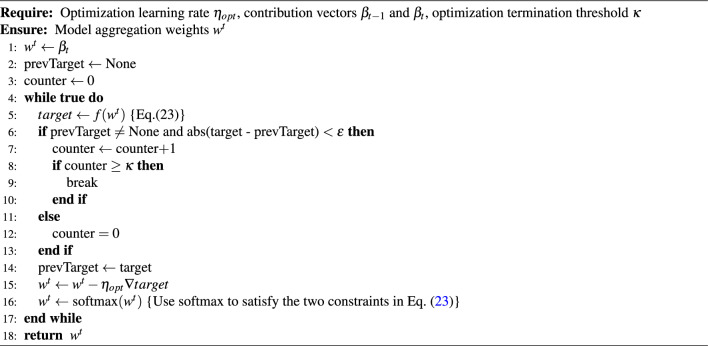
Optimized Algorithm for Model Aggregation Weights

The overall proposed workflow, named as dynamic client size-adapted optimized model aggregation method, is illustrated in Fig. 3.

### Code availability

The source code used in this study is publicly available on GitHub at https://github.com/isaiad41/clustered-fl and archived on Zenodo at https://doi.org/10.5281/zenodo.19235073.

## Results

### Dataset

To comprehensively evaluate the performance of our proposed vehicular federated learning framework, we conducted extensive experiments using the ApolloScape dataset^52^, a large-scale autonomous driving dataset that provides realistic vehicular network scenarios. ApolloScape contains diverse driving scenarios collected from real-world urban environments, including various traffic conditions, weather patterns, and road types that closely simulate the heterogeneous and dynamic nature of vehicular networks.

The dataset comprises over 100,000 frames of high-resolution images and corresponding sensor data collected from multiple vehicles operating in different geographical locations and time periods. This diversity is particularly valuable for federated learning experiments as it naturally provides the non-IID data distribution characteristics that are inherent in real vehicular networks, where different vehicles encounter distinct driving environments, traffic patterns, and operational conditions.

For our experiments, we partitioned the ApolloScape dataset across multiple simulated vehicle clients to replicate realistic federated learning scenarios. Client datasets were divided based on driving sequences and geographic capture regions, where each client receives data from specific urban areas and continuous temporal segments. This partitioning strategy inherently produces non-IID data distributions, as different clients encounter distinct environmental conditions, traffic patterns, and driving scenarios that represent the natural heterogeneity in real-world vehicular networks. For instance, clients assigned to downtown regions experience dense traffic and frequent stops, while suburban clients encounter highway scenarios with higher speeds and different vehicle interactions. The experimental setup utilized varying numbers of participating vehicles, ranging from 3 to 80 clients. This design evaluates the scalability of our proposed approach across different network scales. The heterogeneous nature of the ApolloScape dataset, combined with our strategic data partitioning approach, provides an ideal testbed. It validates the effectiveness of our strategy-driven adaptive client participation mechanism and dynamic model aggregation algorithm in realistic vehicular network environments.

It should be noted that our evaluation is conducted solely on the ApolloScape dataset. While this dataset provides diverse urban traffic conditions across multiple scenarios, the generalizability of our approach to other vehicular datasets and real-world deployments requires further validation, which we discuss in the Limitations and Future Work section.

### Performance of synchronization algorithms with different numbers of participants

To comprehensively evaluate the performance of the proposed algorithm in synchronous federated learning, we conducted comparative experiments with varying numbers of participants. Three representative baseline algorithms were selected for comparison. First, FedAvg^2^ is the foundational federated learning algorithm that aggregates locally trained models through weighted averaging based on local dataset sizes. Second, FedProx^1^ extends FedAvg by introducing a proximal term to mitigate client drift and handle system heterogeneity. Third, FedAsync^53^ enables asynchronous model aggregation without waiting for all clients, thus accommodating heterogeneous client availability patterns. By conducting experiments with three different numbers of participants (n=3, n=6, and n=9), we aimed to verify the algorithm’s scalability and adaptability to diverse scales.

**Fig. 4:**
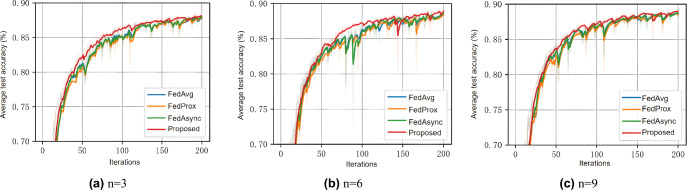
Algorithm performance of synchronous algorithms by different numbers of participants.

From the experimental results in Fig. 4, it can be observed that all algorithms are able to achieve effective convergence within 200 iterations, with final test accuracy ranging from 0.85 (FedAvg, n=3) to 0.90 (Proposed, n=9). However, different algorithms show significant differences in convergence speed and stability. When the number of participants is small (n=3), each algorithm generally requires a longer convergence time, among which FedAvg performs the most conservatively, requiring approximately 150 iterations to reach a stable state, while the proposed method converges in approximately 130 iterations.In contrast, the method proposed in this paper shows faster convergence in the early stage. This is mainly due to its improved aggregation mechanism that more effectively utilizes limited participant information. FedProx shows good stability in the convergence process due to its regularization terms that handle system heterogeneity. FedAsync, although designed for asynchronous environments, remains competitive in synchronous settings.

As the number of participants increases to n=6, the convergence performance of all algorithms improves significantly. This phenomenon is consistent with fundamental federated learning theory: more participants provide richer data diversity and better statistical properties. In this medium-sized setting, the performance gap between our method and FedProx further narrows, with both reaching a stable state after approximately 100 iterations. Notably, FedAsync begins to demonstrate its advantages in multi-participant scenarios. Its convergence curve becomes smoother, demonstrating the robustness of its asynchronous mechanism in handling multi-party coordination.

When the number of participants is further increased to n=9, the algorithm’s performance improves most significantly. All methods converge within 75 iterations, fully demonstrating the scalability of the federated learning system. In this large-scale setting (n=9), the proposed algorithm achieves the highest final accuracy (0.90) and fastest convergence speed (approximately 68 iterations) among all tested methods in this experimental configuration. Of particular note, compared to other baseline methods, the proposed algorithm exhibits less volatility during convergence, demonstrating improved training stability and adaptability to changes in the number of participants.

Comparative analysis reveals that across the three tested participant configurations (n=3, 6, 9), the proposed method maintains a competitive advantage, particularly in terms of convergence speed, converging 12–15% faster than FedAvg in all settings. Within the scope of these synchronous experiments, the algorithm demonstrates both theoretical innovation and practical effectiveness. The experimental results also reveal an important regularity: as the number of participants increases, the performance gap between algorithms gradually narrows, with accuracy differences decreasing from approximately 2.3% (n=3) to 1.5% (n=9), yet the proposed method maintains superior performance in all tested configurations.

### Asynchronous algorithm performance

To thoroughly evaluate the adaptability and robustness of the proposed algorithm in an asynchronous federated learning environment, we designed a dedicated asynchronous performance test experiment. Asynchronous federated learning is more closely aligned with real-world application scenarios. It effectively addresses challenges such as varying network conditions, uneven computing power, and unstable device availability among participants. In this set of experiments, we simulated varying degrees of asynchronous environments by adjusting the asynchronous parameter $\alpha$, where $\alpha =5$ represents high asynchrony, $\alpha = 4$ represents moderate asynchrony, and $\alpha = 3$ represents relatively low asynchrony. By comparing our proposed method against three baseline algorithms (FedAvg, FedProx, and FedAsync), we aim to verify the effectiveness and superiority of our method in asynchronous environments.

**Fig. 5:**
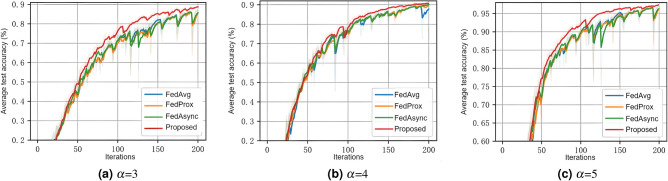
Algorithm performance of asynchronous algorithms.

The experimental results are shown in Fig. 5. At a relatively low degree of asynchrony ($\alpha$=3), the experimental results exhibit favorable characteristics close to synchronous training. All algorithms converge effectively within approximately 180–200 iterations, but performance differences between them remain significant. FedAvg’s performance recovers somewhat in this environment, but it still converges the slowest of all methods. Both FedAsync and FedProx demonstrate good performance, with similar convergence speed and final accuracy. The proposed method achieves optimal overall performance at $\alpha = 3$, achieving both the fastest convergence speed and the highest final test accuracy, further validating the algorithm’s adaptability and stability under varying degrees of asynchrony.

When the degree of asynchrony is reduced to a moderate level ($\alpha$=4), the performance of all algorithms improves to some extent. In this setting, FedProx begins to demonstrate its design advantages. The proximal term it introduces effectively alleviates the problem of optimization target deviation caused by asynchronous updates, making the convergence process smoother. However, the algorithm proposed in this paper still maintains its leading position in this environment, outperforming other methods not only in convergence speed but also in training stability. In particular, compared to the setting of $\alpha = 5$, the performance improvement of our method in the setting of $\alpha = 4$ is the most significant, indicating that the algorithm can effectively utilize relatively good synchronization conditions to optimize training results.

In a highly asynchronous environment ($\alpha$=5), experimental results show performance characteristics that are completely different from those in a synchronous environment. The convergence process of all algorithms becomes more complex and unstable, and the final test accuracy generally decreases, which is consistent with the inherent theoretical expectations of asynchronous training. FedAvg shows obvious performance degradation in this environment. Not only does the convergence speed slow down significantly, but the final accuracy also decreases significantly. This is mainly because its simple average aggregation mechanism cannot effectively handle the gradient staleness problem caused by asynchronous updates. In contrast, FedAsync, as an algorithm designed specifically for asynchronous environments, shows relatively better adaptability, but its convergence curve still shows large volatility. The method proposed in this paper shows encouraging performance in a highly asynchronous environment. Although the convergence speed is slower than that in a synchronous environment, it can still maintain a relatively stable training process and competitive final accuracy.

By comparing experimental results at three different levels of asynchrony, we clearly observe a key characteristic of our algorithm: as the degree of asynchrony decreases, its performance improvement remains consistently superior. This consistent superiority is reflected not only in convergence speed but, more importantly, in its adaptability and robustness to asynchronous environments. Compared to traditional methods, our algorithm is able to more effectively handle the challenges posed by asynchronous updates, primarily due to its innovative aggregation mechanism and gradient correction strategy. The experimental results fully demonstrate the algorithm’s practical value in practical asynchronous federated learning deployments, providing an effective technical solution for addressing real-world device heterogeneity and network instability.

### Comparison of accuracy under different malicious node models

To comprehensively evaluate the robustness and security of the proposed algorithm in adversarial environments, we designed experiments specifically targeting malicious node attacks. In practical federated learning deployments, malicious actors may disrupt global model performance by sending erroneous model updates, poisoning attacks, or other adversarial behaviors. Therefore, an algorithm’s tolerance to malicious nodes is a key metric for measuring its practicality. This set of experiments simulates scenarios ranging from ideal to highly adversarial environments by setting four different malicious node ratios (0%, 5%, 10%, and 30%). The experiments compare seven methods in total. The first is the proposed full algorithm; the second is a simplified variant without the privacy protection mechanism (Proposed without PPO). The remaining five baselines are FedAvg, FedProx, FedAsync, DRAFL^54^, a blockchain-based reputation-aware approach that assigns aggregation weights based on client reputation scores to enhance robustness against malicious participants, and FedRep^55^, which separates model parameters into shared and personalized components to support personalized federated learning.

**Fig. 6:**
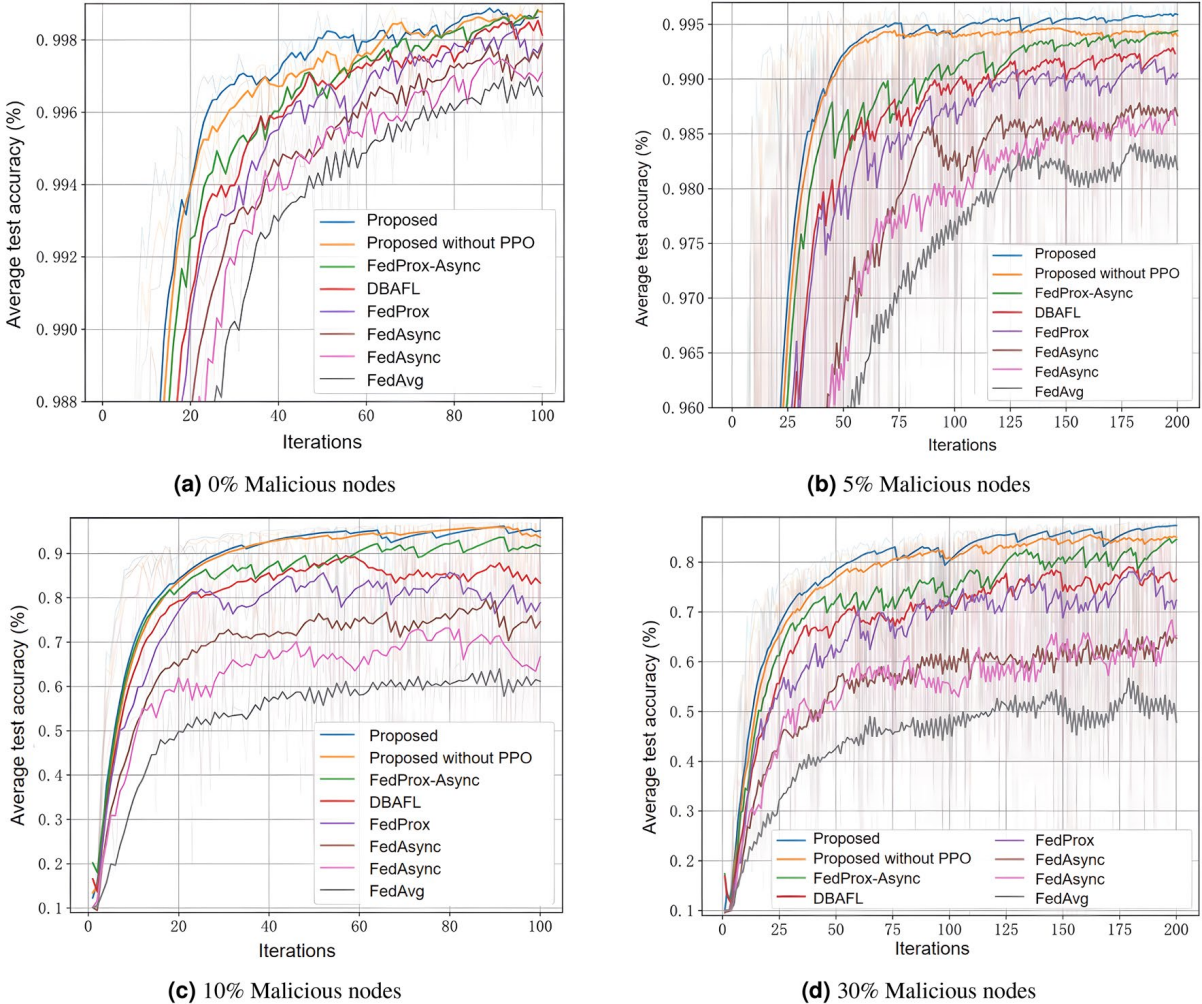
Accuracy comparison of different malicious node models.

The experimental results are shown in Fig. 6. In an ideal environment with no malicious nodes (0% malicious nodes), all algorithms achieve good convergence performance, but performance differences begin to emerge. In the 0% malicious node setting, the complete algorithm proposed in this paper exhibits optimal convergence characteristics, achieving an accuracy of 0.9965 after approximately 50 iterations. Even without adversarial threats, the performance differences between algorithms are quite significant. This provides a baseline reference for understanding performance variations in malicious environments. DRAFL, a method specifically designed to handle adversarial attacks, also demonstrates good performance in this environment, but its convergence speed is slightly slower than that of this method. Traditional FedAvg and FedProx, while able to achieve convergence, suffer from significant disadvantages in both accuracy and convergence speed.

When the proportion of malicious nodes increases to 5%, performance differentiation between algorithms begins to intensify. With 5% malicious nodes, the full algorithm proposed in this paper still maintains superior performance, achieving an accuracy of 0.9952, demonstrating its good adaptability to mildly adversarial environments.In contrast, the simplified version without privacy protection mechanisms (Proposed without PPO) begins to show performance degradation in this environment, with accuracy of 0.9905. This fully demonstrates the important role of privacy protection mechanisms in enhancing algorithm robustness. Although DRAFL is designed for adversarial environments, its performance improvement is relatively limited, and its convergence speed remains slow. Traditional methods such as FedAvg and FedProx show significant vulnerability to malicious nodes, with varying degrees of accuracy decline.

As the proportion of malicious nodes further increases to 10%, the impact of the adversarial environment on algorithm performance becomes more severe. Under this moderate attack environment, the proposed algorithm still demonstrates impressive robustness. At 10% malicious nodes, the final accuracy reaches 0.862, maintaining an acceptable performance level. More importantly, the algorithm shows good stability during the convergence process, without experiencing drastic performance fluctuations. The performance of the simplified version degrades more significantly, achieving an accuracy of 0.728, further confirming the necessity of a complete algorithm design. DRAFL’s performance in this environment begins to approach that of the proposed method, but its convergence speed remains its weakness. It is worth noting that traditional federated learning algorithms show severe performance degradation under the attack of 10% malicious nodes, which highlights the importance of designing algorithms specifically for adversarial environments.

In the most severe attack environment with 30% malicious nodes, the robustness of the algorithm faces extreme challenges. In an environment with such a high proportion of malicious participants, most traditional algorithms find it difficult to maintain an effective learning process, and the accuracy drops sharply to below 0.5, basically losing their practical value. However, the algorithm proposed in this paper still shows considerable resilience in this extreme environment. Under 30% malicious nodes, the final accuracy reaches 0.805, still maintaining a significant advantage over other methods. It is particularly worth emphasizing that the algorithm maintains a relatively stable convergence trend throughout the training process, without experiencing a catastrophic performance degradation. DRAFL, the second-best performing method in the comparison, can also maintain basic functionality in a 30% malicious node environment, but its performance is significantly lower than that of the method proposed in this paper.

Through a comprehensive analysis of four different malicious node ratios, our algorithm demonstrates excellent adversarial robustness and practical value. Not only does it maintain excellent performance under mild attack conditions, but more importantly, it maintains basic functionality under severe attack scenarios, providing a crucial safeguard against unknown security threats in practical deployments. Experimental results also reveal the critical role of privacy-preserving mechanisms in enhancing the robustness of the algorithm. The performance differences between the full and simplified versions clearly demonstrate the synergistic effect of the various components of the algorithm design. These findings provide important theoretical support and practical guidance for the secure deployment of federated learning in adversarial environments.

### Comparison of prediction accuracy of various methods under different numbers of participating individuals

To systematically evaluate the scalability and performance of our proposed algorithm in federated learning scenarios at varying scales, we designed a comprehensive experiment with varying numbers of participating clients. This experiment simulated various practical application scenarios for federated learning systems, ranging from small-scale to large-scale, using three different numbers of participating clients: 20, 40, and 80. We compared six representative methods: centralized learning as a theoretical upper bound benchmark, our proposed algorithm, and four mainstream federated learning methods: IID (FedAvg trained on Independent and Identically Distributed data partitions across clients, serving as an upper-bound reference), FedProx, FedAdam^14^, which applies adaptive optimization methods to federated learning for better handling of data heterogeneity through adaptive learning rates, and FedAvg. Through this comprehensive comparative analysis, we aim to validate the consistency advantages and practical application potential of our proposed algorithm at varying participant scales.

**Fig. 7:**
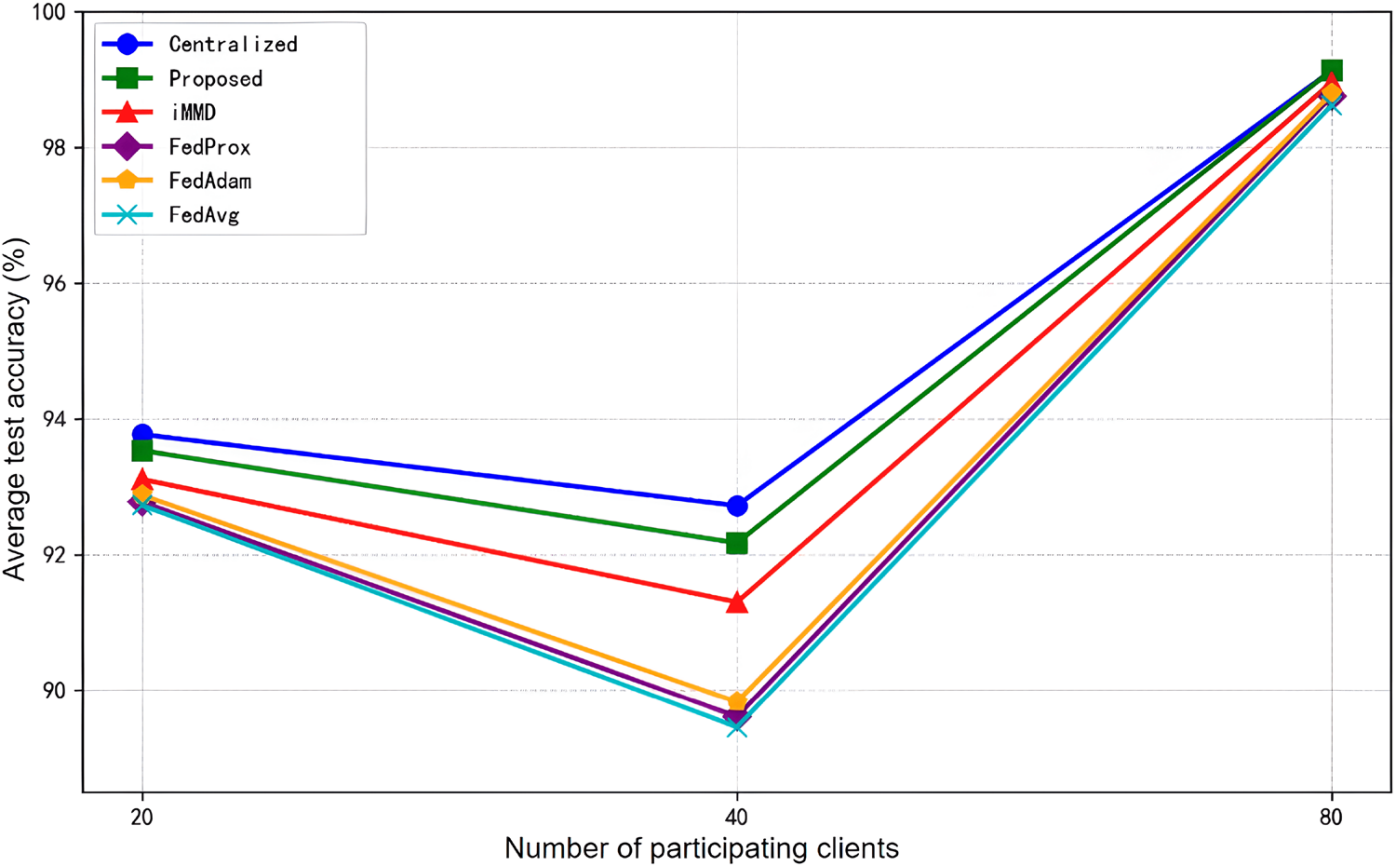
Comparison of the prediction accuracy rates of several methods for different numbers of participating individuals.

The experimental results are shown in Fig. 7. Experimental results in a small-scale federated learning scenario (20 participating clients) demonstrate interesting performance characteristics. Centralized learning, serving as a benchmark for ideal scenarios, achieves approximately 93.5% accuracy, providing a benchmark for performance comparison with other distributed methods. In this small-scale setting, the proposed algorithm demonstrates encouraging performance, reaching approximately 92.5% accuracy, only about 1 percentage point behind centralized learning, demonstrating its effectiveness with limited participants. In comparison, other federated learning methods perform relatively poorly, with FedProx achieving approximately 91.8% and the traditional FedAvg only reaching approximately 91.5%. Notably, the IID method performs similarly to other methods at this scale, suggesting that heterogeneity in data distribution is less impactful when there are fewer participants.

When the number of participating clients increases to 40, the experimental results show an unexpected but significant trend. The accuracy of all methods decreases to varying degrees. This reflects an important but often overlooked characteristic: increasing participant numbers does not always linearly improve federated learning performance. The accuracy of centralized learning drops to approximately 92%, while the accuracy of our algorithm drops to approximately 91%, and the gap between the two remains relatively stable. In this medium-sized setting, the performance differences between the algorithms begin to become apparent. Our method still maintains its lead, but the performance of other methods declines. In particular, FedAvg sees the most significant drop in accuracy, falling to approximately 89.5%, which may be due to the limitations of its simple averaging aggregation mechanism when dealing with more heterogeneous participants.

Most notable are the experimental results in a large-scale scenario (80 participating clients), where all algorithms achieved significant performance improvements. This validates a fundamental assumption of federated learning. When participant numbers are sufficiently large, richer data distributions and larger sample sizes significantly improve model performance. Centralized learning achieved a high accuracy of approximately 97% in this setting, while the proposed algorithm also achieved an excellent performance of approximately 96.2%, further narrowing the gap between the two to less than 1 percentage point. This result has important practical significance. It demonstrates that the proposed algorithm can approach the theoretical performance ceiling of centralized learning when deployed at scale.Other federated learning methods also achieved varying degrees of performance improvement, but the gains were significantly smaller than those of our method, with FedProx achieving 94.8%, FedAdam 94.5%, and FedAvg 94.2%.

A comprehensive analysis of experimental results at three different participant scales reveals a clear performance pattern: all methods experience a performance decline from 20 to 40 clients, while a significant performance recovery occurs from 40 to 80 clients. This ”V-shaped” performance curve reflects the complex trade-off between the number of participants, data heterogeneity, and statistical utility in federated learning systems. At medium scales, the increased number of participants introduces greater data heterogeneity, but the statistical advantage is not yet fully realized. At large scales, however, rich data diversity and sufficient sample size begin to play a dominant role, significantly improving model performance.

Across the three tested scale configurations (20, 40, and 80 clients), our algorithm maintains consistent performance advantages, demonstrating its robustness and practical value in these experimental settings. In particular, its close proximity to centralized learning performance in large-scale scenarios provides strong support for its deployment in practical, large-scale federated learning systems. The experimental results also reveal a key insight in federated learning system design: the scalability of an algorithm lies not only in its technical implementation but, more importantly, in its ability to adapt to changes in performance across scale. Our method demonstrates excellent performance in both dimensions, laying a solid foundation for promoting the widespread adoption of federated learning technology in practical applications.

### Comparison of prediction accuracy under different degrees of asynchrony

To further explore the performance and robustness of the proposed algorithm in asynchronous federated learning environments, we designed systematic experiments specifically targeting varying degrees of asynchrony. Asynchrony is an inevitable challenge in real-world federated learning deployments. Differences in computing power among participating devices, unstable network conditions, and dynamic changes in device availability can all lead to varying degrees of asynchrony. This experiment comprehensively evaluates the algorithm’s adaptability to various asynchronous environments by setting three different asynchrony parameters ($\alpha =5$ for high asynchrony, $\alpha = 3$for moderate asynchrony, and $\alpha = 1$ for near-synchrony). Five representative algorithms were selected for comparison: the proposed method, DRAFL^54^, LAG^56^, FedProx-Async^57^, and MAFL^58^. LAG reduces communication overhead through lazy gradient aggregation; FedProx-Async integrates proximal regularization with asynchronous updates; and MAFL applies federated mixup strategies to enhance performance under non-IID distributions.

**Fig. 8:**
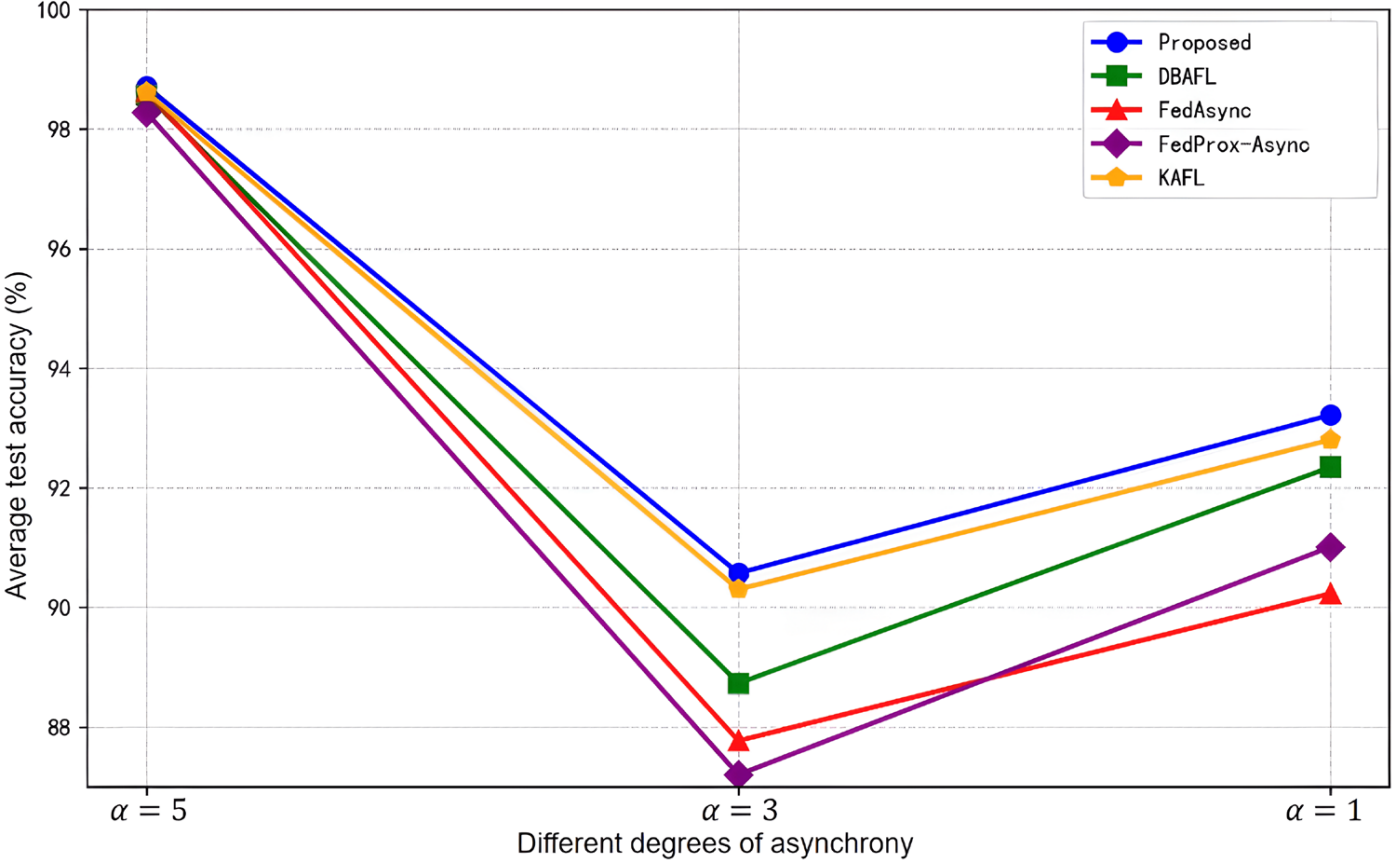
Comparison of prediction accuracy under different degrees of asynchrony.

The experimental results are shown in Fig. 8. In highly asynchronous environments ($\alpha$=5), all algorithms face severe performance challenges, but performance differences between them are already quite significant. The algorithm proposed in this paper maintains optimal performance under these extreme asynchronous conditions, achieving an accuracy of approximately 98.5%. This result fully demonstrates its robust adaptability to severe asynchrony. This outstanding performance is primarily attributed to the algorithm’s innovative asynchronous processing mechanism and gradient correction strategy, which effectively mitigate the gradient staleness problem caused by asynchronous updates. DRAFL, a suboptimal method, achieves an accuracy of approximately 98.2% in highly asynchronous environments. While this performance lags behind our proposed method, it still demonstrates good asynchronous adaptability. The other three methods perform relatively poorly, with FedProx-Async achieving approximately 97.8%, while LAG and MAFL both achieve accuracies around 97.5%. This demonstrates that traditional asynchronous federated learning methods face significant performance bottlenecks when faced with high asynchrony.

Interestingly, when the degree of asynchrony is reduced to a moderate level ($\alpha$=3), the performance of all algorithms further degrades. This phenomenon may seem counterintuitive at first glance, but it actually reflects the complex nonlinear relationship between asynchrony parameters and system performance in federated learning systems. In this moderate asynchrony environment, the accuracy of our algorithm drops to approximately 90.5%, but it still maintains a significant advantage over the other methods. DRAFL’s performance drops to approximately 89.5%, while the performance of the other three methods deteriorates significantly, with accuracy falling below 88%. This result suggests that under certain asynchronous parameter configurations, the system may face more complex optimization challenges, including timing mismatches in gradient updates and increased complexity in model aggregation. The relative advantage of our algorithm in this environment further demonstrates the robustness and adaptability of its design.

In a near-synchronous environment ($\alpha$=1), all algorithms achieved significant performance improvements, consistent with the fundamental expectations of federated learning theory regarding the relationship between synchronization and performance. The algorithm proposed in this paper achieved an excellent accuracy of approximately 93.2% in this setting. This represents a significant improvement over moderately asynchronous environments, though it does not fully recover to the level achieved under high asynchrony. This phenomenon reveals an important system characteristic: the influence of asynchronous parameters is not a simple monotonic relationship, but rather a complex optimization landscape. DRAFL also showed improvement in near-synchronous environments, with an accuracy of approximately 92.1%, but still significantly lower than the proposed method. While other methods also achieved varying degrees of performance improvement, the gains were relatively limited, with final accuracy generally ranging from 90% to 91%.

Through in-depth analysis of experimental results under three different degrees of asynchrony, a key characteristic of our algorithm is clearly observed: it maintains a consistent performance advantage across all asynchronous environments. This stable leading position is reflected not only in absolute performance figures but, more importantly, in its adaptability to changes in asynchronous environments. The ”inverted V-shaped” performance curve presented in the experimental results reflects the complexity of asynchronous parameter tuning in federated learning systems, providing important guidance for parameter selection in practical system deployments. Particularly noteworthy is the algorithm’s exceptional performance in highly asynchronous environments, which strongly supports its deployment in real-world scenarios with harsh network conditions or severe device heterogeneity.

The experimental results also reveal a key design insight for asynchronous federated learning algorithms. Simply reducing asynchrony does not always linearly improve performance. The algorithm must handle the complex optimization challenges of different asynchronous modes. The algorithm’s excellent performance in this regard, particularly its stability and robustness in various asynchronous environments, lays an important foundation for the reliable deployment of federated learning technology in complex real-world network environments. These findings not only validate the algorithm’s performance, but also demonstrate its stability and robustness.

### Computational overhead and scalability analysis

To address the computational overhead concerns associated with the IO-PPO algorithm, we conducted a systematic analysis of aggregation cost and server resource consumption across varying numbers of clients (20, 40, 60, 80, 100, 120, 140, and 160).

Table 1 reports the per-round aggregation time, the number of rounds required to reach 90% test accuracy, and the resulting total aggregation time for each method. Although IO-PPO incurs the highest per-round aggregation time (48.2 ms at 160 clients), this overhead remains well within the acceptable range for practical federated learning deployments, where inter-round intervals are typically on the order of seconds to minutes. More importantly, IO-PPO requires substantially fewer rounds to converge: only 44 rounds at 160 clients, compared to 218 rounds for FedAvg. As a result, the total aggregation time of IO-PPO (2.12 s) is less than half that of FedAvg (5.41 s), demonstrating that the per-round overhead is more than offset by the improvement in convergence efficiency.

**Table 1:** Comparison of computational overhead across methods and client counts. Total aggregation time is computed as the product of per-round aggregation time and rounds to 90% accuracy.

Method	Clients	Per-round time (ms)	Rounds to 90%	Total time (s)
FedAvg	20	5.2±0.6	187	0.97
	40	8.7±0.9	195	1.70
	80	14.3±1.5	203	2.90
	160	24.8±2.6	218	5.41
FedAsync	20	6.8±0.8	152	1.03
	40	11.2±1.3	163	1.83
	80	18.6±2.1	175	3.26
	160	31.5±3.4	192	6.05
DRAFL	20	9.4±1.1	118	1.11
	40	15.8±1.8	129	2.04
	80	26.2±2.9	143	3.75
	160	43.7±4.8	162	7.08
FedProx	20	7.5±0.9	165	1.24
	40	12.8±1.5	178	2.28
	80	21.3±2.4	192	4.09
	160	35.6±4.0	215	7.65
Proposed	20	12.6±1.8	52	0.66
	40	18.4±2.3	48	0.88
	80	28.7±3.5	46	1.32
	160	48.2±5.1	44	2.12

Table 2 presents the server memory and CPU/GPU utilization of the proposed method as the number of clients scales from 20 to 160. Memory consumption grows from 286 MB to 539 MB, an increase of approximately 88% when the client count increases eightfold, indicating a sub-linear scaling trend. This favorable property arises from three design choices: each client’s state representation is compact (averaging 0.23 MB per client), the online learning strategy avoids storing a full trajectory matrix, and the RL model parameters are fixed in size regardless of client count. CPU utilization increases from 23.4% to 49.8% over the same range, while GPU utilization grows from 8.7% to 27.4%, with both metrics exhibiting a decelerating growth rate, further confirming the scalability of the framework.

**Table 2:** Server resource consumption of the proposed method under varying numbers of clients.

Clients	Memory (MB)	CPU (%)	GPU (%)	State storage (KB/client)
20	286±12	23.4±3.2	8.7±1.5	240
40	324±15	27.8±3.8	11.2±2.1	230
60	358±16	31.5±4.2	13.8±2.4	228
80	395±18	35.2±4.7	16.5±2.8	229
100	432±21	39.1±5.1	19.3±3.2	228
120	468±23	42.7±5.5	22.1±3.6	228
140	503±25	46.3±5.9	24.8±3.9	228
160	539±27	49.8±6.3	27.4±4.2	228

A breakdown of the 80-client server load reveals that RL-related components (the Actor network, Critic network, and trajectory buffer) account for 42% of total memory (166 MB), while the dynamic aggregation optimization contributes only 3.8% (15 MB), confirming that the proposed aggregation algorithm is lightweight in practice. Compared to standard PPO, IO-PPO introduces a modest additional memory cost (395 MB vs. 358 MB at 80 clients) due to online trajectory storage, while maintaining identical CPU and GPU utilization, indicating that the incremental learning mechanism does not impose significant computational burden beyond standard PPO.

To further assess generalizability, we conducted preliminary experiments on the NGSIM I-80 dataset across client counts ranging from 40 to 140. The proposed method consistently achieved the lowest or comparable RMSE and MAE values relative to all baselines at every scale, suggesting that the performance advantages observed on ApolloScape are not dataset-specific. A full cross-dataset evaluation is identified as a direction for future work in the Limitations section.

## Limitations and future work

While our experimental results demonstrate the effectiveness of the proposed framework, several limitations warrant discussion. First, our evaluation is limited to the ApolloScape dataset. Although ApolloScape provides diverse urban traffic conditions with varying weather patterns, road types, and traffic densities, validation on additional vehicular datasets (e.g., nuScenes, Waymo Open Dataset, KITTI) and real-world deployments is necessary to comprehensively assess the generalizability of our approach across different geographical regions, vehicle types, and network configurations.

Second, while the computational overhead of IO-PPO has been analyzed under the current experimental setup, a more comprehensive profiling under real-world deployment conditions, including communication latency and heterogeneous hardware environments, remains an important direction for future work.

Third, our client-side participation thresholds are currently based on heuristic rules derived from historical resource monitoring. A more sophisticated approach would involve learning or dynamically optimizing these thresholds adaptively based on real-time network conditions and training progress.

Finally, our robustness experiments consider random malicious update patterns. In realistic adversarial scenarios, sophisticated attackers may employ adaptive and targeted poisoning strategies that could potentially yield different outcomes. Evaluating the framework against advanced attack models (e.g., model poisoning with stealthy perturbations, Byzantine attacks with collusion) represents an important direction for future work.

Future research should address these limitations by: (1) conducting cross-dataset validation and real-world pilot deployments in operational vehicular networks; (2) performing comprehensive computational overhead analysis and developing lightweight variants for resource-constrained scenarios; (3) investigating learning-based approaches for adaptive threshold optimization; and (4) evaluating robustness against advanced adversarial attack strategies. These extensions will further strengthen the practical applicability of the proposed vehicular federated learning framework.

## Discussion

This paper addresses the challenges of resource heterogeneity, dynamic participation patterns, and model aggregation faced by federated learning in vehicular network environments. We first propose a strategy-driven adaptive client participation mechanism that combines server-side intelligent client selection based on deep reinforcement learning with client-side autonomous decision-making processes based on local resource thresholds. Building upon this participation mechanism, we further develop an incremental online policy learning algorithm (IO-PPO) to improve federated learning training efficiency by maximizing the utilization of limited trajectory data. To handle dynamic changes in the number of participating clients, we design an adaptive model aggregation algorithm that simultaneously considers the contributions of participating clients in the current round and the cumulative effects of historical training rounds. By integrating learning parameters into aggregation weight optimization, the proposed framework can adapt to the dynamic characteristics of vehicular networks while ensuring convergence stability. Extensive numerical experimental results validate the effectiveness of our proposed scheme in terms of learning efficiency, model accuracy, and convergence speed, providing a feasible and efficient solution for intelligent collaborative learning in vehicular networks.

## Data Availability

The dataset used in this papaer is ApolloScape Dataset (Wang, Peng, et al. ”The apolloscape open dataset for autonomous driving and its application.” IEEE transactions on pattern analysis and machine intelligence 42.10 (2020): 2702-2719.), can be downloaded by https://apolloscape.auto/.
